# Transcriptome profiling of abiotic responses to heat, cold, salt, and osmotic stress of *Capsicum annuum* L.

**DOI:** 10.1038/s41597-020-0352-7

**Published:** 2020-01-13

**Authors:** Won-Hee Kang, Young Mi Sim, Namjin Koo, Jae-Young Nam, Junesung Lee, Nayoung Kim, Hakgi Jang, Yong-Min Kim, Seon-In Yeom

**Affiliations:** 1 Institute of Agriculture & Life Science, Gyeongsang National University, Jinju, 52828 Republic of Korea; 2 Korean Bioinformation Center, Korea Research Institute of Bioscience and Biotechnology, Daejeon, 34141 Republic of Korea; 3 Department of Agricultural Plant Science, Division of Applied Life Science (BK21 Plus Program), Gyeongsang National University, Jinju, 52828 Republic of Korea

**Keywords:** Plant stress responses, Transcriptomics, RNA sequencing, Agriculture

## Abstract

Peppers (*Capsicum annuum* L.), belonging to the Solanaceae family, are one of the most economically important crops globally. Like other crops, peppers are threatened by diverse environmental conditions due to different pathogens and abiotic stresses. High-quality reference genomes with massive datasets of transcriptomes from various conditions can provide clues to preferred agronomic traits for breeding. However, few global gene expression profiling datasets have been published to examine the environmental stress-resistant mechanisms in peppers. In this study, we report the RNA-seq analyses of peppers treated with heat, cold, salinity, and osmotic stress at six different time points. RNA-seq libraries from 78 RNA samples containing three biological replicates per time point for each of the abiotic stresses and a mock control were constructed. A total of 204.68 Gb of transcriptome data were verified by differentially expressed genes and gene ontology enrichment analysis. Analyses of the transcriptome data in this study will provide useful information for basic studies of various stimuli to facilitate the development of stress-resistant pepper cultivars.

## Abstract (summary)

**Table:** 

Measurement(s)	RNA • transcriptome
Technology Type(s)	RNA sequencing
Factor Type(s)	time point measurement
Sample Characteristic - Organism	Capsicum annuum

Machine-accessible metadata file describing the reported data: 10.6084/m9.figshare.11440659

## Background & Summary

Abiotic stresses, such as heat, cold, drought, and salinity, which affect the condition of the soil can decrease crop quality, reduce crop production, and threaten food security. Plants respond to abiotic stresses via dynamic and complex reactions that accompany molecular, cellular, and physiological changes in plant tissues^1^. To understand the responses of plants to abiotic stresses, diverse crop breeding approaches have been applied from traditional breeding methods to variable -omics methods, such as next-generation sequencing (NGS).

Since the development of NGS, the transcriptome has been widely studied to gain insights into the molecular mechanisms by which plant species adapt to their environment. Currently, transcriptome data analyses of plants are performed in various organisms under diverse conditions, including exposure to abiotic stresses. Most transcriptome studies involving abiotic stresses have been performed in model plants, with a few studies examining crops treated with one or two different stresses at a certain plant development stage^2–8^. Therefore, limited comparative transcriptome analyses for plants responding to different abiotic stresses have been performed.

Peppers (*Capsicum* spp.) are an economically important crop belonging to the Solanaceae family. Over the last two decades, the production and cultivation of chili peppers have steadily increased worldwide, reaching 3.8 million ha of land for cultivation and 40.7 million tons of peppers produced in 2017 (FAO; www.fao.org). Recently, multiple reference pepper genomes and transcriptomes have been published; these datasets can be used to obtain abundant information on pepper breeding traits^9–11^. However, few studies have examined pepper breeding in diverse conditions, such as during exposure to abiotic and biotic stresses. Comprehensive transcriptome analyses under such diverse conditions are required to obtain a wide variety of gene expression profiles and identify complex gene expression networks.

In this study, we present transcriptome analyses of peppers subjected to four major environment stresses—heat, cold, drought, and salinity—at the same time points and at the same plant stages. We describe in detail the construction of 78 RNA-seq libraries for heat-, cold-, mannitol-, and NaCl-treated and untreated control samples at 0, 3, 6, 12, 24, and 72 h. A total of 204.68 Gb of transcriptome data were generated using transcriptome analysis pipelines consisting of quality control, quantification, and differential gene expression analyses. A principal components analysis (PCA) test, hierarchical clustering of gene expression data and gene ontology (GO) enrichment analysis were used to infer the quality of the RNA-seq data and the characteristics of samples in each treatment. The extensive transcriptome data obtained will provide valuable information for future studies of crops exposed to abiotic stresses.

## Methods

### Overview of experimental design

**Fig. 1:**
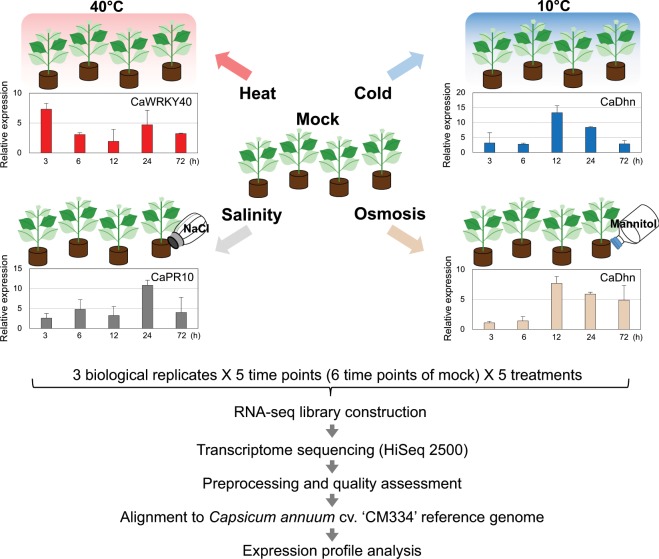
Overview of experimental design and analysis pipeline. RNA from pepper leaves subjected to each abiotic stress (heat, cold, salinity, and osmotic stress) and the 0-h sample from the mock control was harvested. Marker gene expression was confirmed for each stress condition, and the values were normalized to *C. annuum* actin expression and were calculated relative to control group as mean values with standard deviation. The validated RNAs were sequenced by the Illumina HiSeq 2500 system. All RNA-seq reads were preprocessed for a quality assessment. The filtered transcriptome reads were aligned to the CM334 genome, and the expression profile was analyzed.

The third or fourth leaves were collected from four pepper plants per each biological replicate. Leaves were harvested at 3, 6, 12, 24, and 72 h after treatment. Mock controls were simultaneously collected with each abiotic treatment sample at 0, 3, 6, 12, 24, and 72 h. Marker gene expression for each condition was confirmed for 78 RNA samples by RT-PCR (Fig. 1). Subsequently, RNA-seq libraries were constructed and sequenced. Transcriptome data were used to conduct a quality assessment and aligned to *Capsicum annuum* cv. CM334 reference genome (v.1.6). The workflows for the abiotic stress treatment and transcriptome data analysis pipeline are presented in Fig. 1.

### Plant materials and treatment

Two weeks after germination, the pepper seedlings were transplanted into a 32-plug tray (6 cm in diameter by 6.5 cm in height) and maintained in a growth room at 24 ± 1 °C with a 16-h light and 8-h dark photoperiod. At the six-true-leaf stage, plants were subjected to a temperature of 10 °C or 40 °C to mimic cold or heat stress, respectively. For salinity stress, plants were treated with 50 mL of a 400 mM NaCl solution; for osmotic stress, the peppers were treated with 50 mL of 400 mM mannitol. For transcriptome profiling, the third or fourth leaves from four plants were harvested per replicate at 0, 3, 6, 12, 24, and 72 h after treatment (Fig. 1). Three biological replicates at each time point per condition were collected. The leaf samples were flash-frozen in liquid nitrogen and stored at −80 °C until RNA isolation.

### RNA extraction, library construction, and sequencing

Total RNA was extracted from pepper leaf samples (100 mg) using the Trizol reagent (Ambion, USA), according to the manufacturer’s instructions. To perform RNA quality control, RNA was quantified spectrophotometrically using a NanoDrop 2000 spectrophotometer (Thermo Scientific, USA), and RNA integrity was verified by agarose gel electrophoresis. Marker gene expression for each treatment was confirmed by RT-PCR analysis using primers specific for each marker gene: heat stress (*CaWRKY*)^12^, cold stress (*CaDhn*)^13^, salinity stress (*CaPR10*)^14^, and osmotic stress (*CaDhn*)^13^ (Fig. 1). RT-PCR was performed using a GeneAtlas thermo-cycler G-02 device (Astec, Japan) using rTaq DNA polymerase (Elpis, Korea) as described by the manufacturer. The gene expression level was normalized to the expression of the *CaActin* gene and was calculated relative to mock control. Values were calculated following three replications with standard deviations (Fig. 1). Five micrograms of each RNA sample were used to generate a strand-specific library containing inserts of approximately 150–200 bp in size, as previously described^15^. In total, 78 cDNA libraries from five treatments (i.e., the four abiotic stresses and a mock control) were constructed for transcriptome analysis (Table 1). For RNA sequencing, 150-nt, paired-end sequencing was conducted using a HiSeq 2500 platform (Illumina, USA) at Macrogen (Korea).

**Table 1:** Statistical summary of RNA-seq data used in this study.

Treatment	Time point	Read type	Read length (bp)	Processed read length (bp)	Processed data (Gb)	Accession number
Mock	0, 3, 6, 12, 24, 72 h	Paired	151	145.56	45.53	SRP187794
Cold	3, 6, 12, 24, 72 h	Paired	151	144.36	40.25	
Heat	3, 6, 12, 24, 72 h	Paired	151	145.50	35.77	
Mannitol	3, 6, 12, 24, 72 h	Paired	151	146.50	39.11	
NaCl	3, 6, 12, 24, 72 h	Paired	151	145.89	32.68	

### Data preprocessing, gene quantification, and GO enrichment analysis

The raw RNA sequences were filtered and trimmed using cutadapt^16^ and the NGS QC Toolkit^17^ to remove low-quality bases and adapter sequences. After filtering, the trimmed reads were assessed using FastQC (https://www.bioinformatics.babraham.ac.uk/projects/fastqc/), and quality results were then merged with multiQC^18^ using default parameters. The preprocessed reads were aligned to *C. annuum* v.1.6 reference genome (GenBank Accessions: AYRZ00000000)^19^ and the annotation gene model v.2.0 (http://peppergenome.snu.ac.kr) using Hisat v2-2.1.0 software^20^ and default parameters. Transcriptome quantification was performed using featureCounts^21^ to calculate the transcript read counts. Raw read counts were normalized using TMM methods^22^. PCA was performed using previously published code with modification^23^.

We used the edgeR with three package (estimateDisp, glmQLFit, and glmQLFTest) to conduct differential expression analysis^24^. To design a model formula, all the experimental factors such as time points and treatments were combined into one factor. Then, we found genes that respond differently between the treatment and the mock at any time points. Genes with an adjusted p-value < 0.05 and fold change (FC) > |2| were considered differentially expressed genes (DEGs). The DEGs for each point in treatments were collected to union sets of the DE gene across the time points. The top 30 genes of DEGs for each stress were displayed into heatmap using pheatmap package^25^. GO enrichment analysis for DEGs were performed based on the functional annotation of Arabidopsis genome (TAIR10, http://www.arabidopsis.org). The best hit proteins were mapped by the best match of BLASTP with filtering category (query coverage ≥60%, subject coverage ≥60% and identity ≥60%). The GO enrichment analysis for best hit proteins was performed using clusterProfiler^26^ in the R package with org.At.tair.db for Arabidopsis annotation package^27^ (See the file “Programs and code information.docx” at figshare). The enriched GO terms were obtained by FDR < 0.05 and minGSize = 10. Then, the stress-related GO terms were visualized using ggplot2^28^. All expression profiling, DEG information and the results of GO enrichment analysis are available at figshare^27^ (see the files “Normalized TMM.zip”, “DEG result for each stress.zip” and “GO enrichment analysis result.zip” at figshare).

## Data Records

The RNA-seq raw data of 78 samples are deposited at the NCBI Sequence Read Archive (SRA) with identifier SRP187794^29^. The gene expression quantification data of all the samples was deposited at Gene Expression Omnibus (GEO) database with identifier^30^. The combined additional files and information generating this study have been uploaded to figshare^27^.

## Technical Validation

### Quality control

**Fig. 2:**
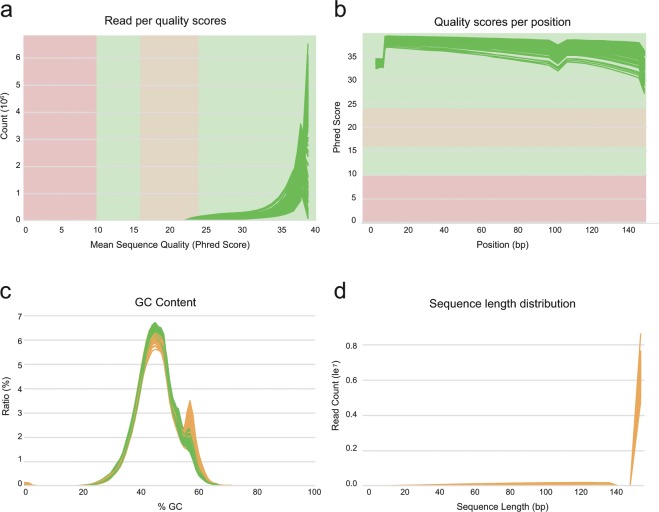
Results of raw read preprocessing. (**a**) Mean quality scores per read. The *x*-axis represents the mean quality scores, and the *y*-axis depicts the read counts. (**b**) Mean quality scores per position. The *x*-axis represents the position, and the *y*-axis depicts the Phred score. (**c**) GC content of reads. The *x*-axis represents the GC content, and the *y*-axis depicts the ratio of reads. (**d**) Distribution of read length. The *x*-axis depicts the sequence length, and the *y*-axis represents the read counts.

The quality of the RNA-seq data was assessed by investigating the mean quality score per position and per sequence, as well as the GC content and read length distribution using FastQC and multiQC^18^. The assessment plots are shown in Fig. 2. The quality scores of the bases per position were higher than the Phred quality score of 25, and all reads were greater than the quality score of 20. The GC content of all samples was shown as a normal distribution; these data indicate a lack of sequence contamination during the sequencing process. These statistics revealed that the raw reads were of high quality. Alignments of the preprocessed reads had a high mapping rate, which on average 70.14% and 90.38% in the gene model (v.2.0) and reference genome of *C. annuum* (v.1.6), respectively^27^ (see the file “Statistical summary of RNA-seq for each sample.xlsx” at figshare).

### Analysis of transcriptome data

**Fig. 3:**
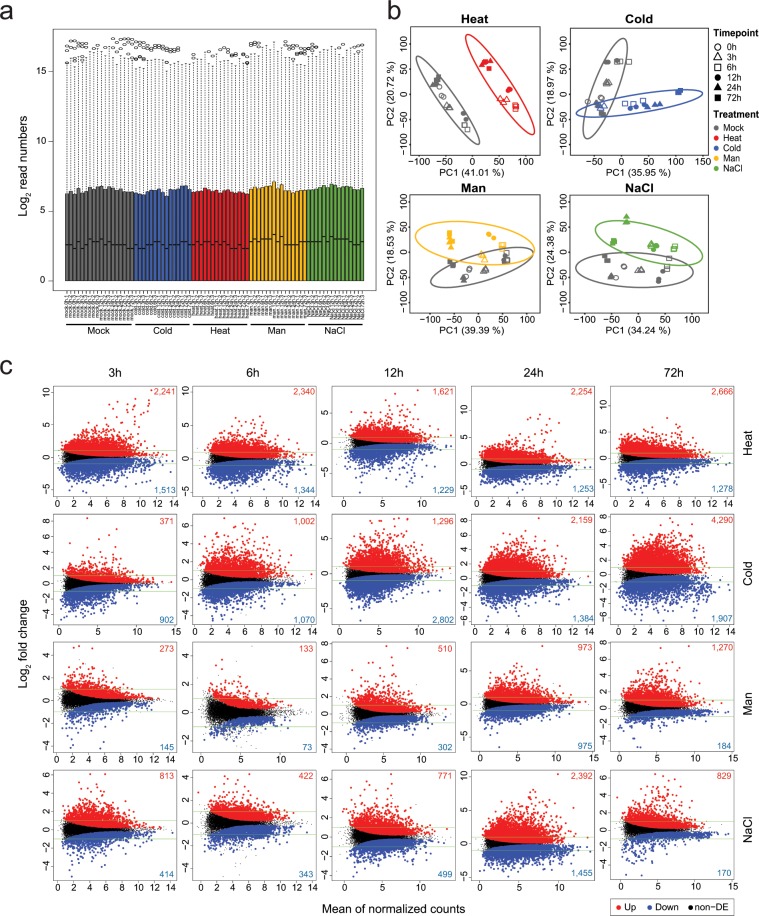
Global assessments of transcriptome data. (**a**) Normalized raw reads. (**b**) Principal components analysis for each stress. (**c**) MD plot of DEGs for each stress. The numbers of up- and down- regulated genes are shown in red and blue in each plot, respectively. Man, mannitol.

**Fig. 4:**
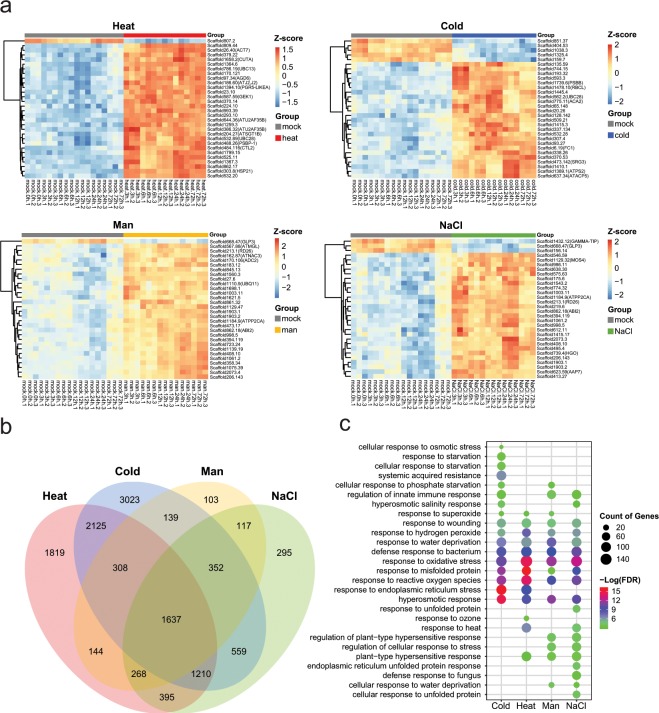
Expression profiles in response to abiotic stresses. (**a**) Expression patterns of top 30 DEGs for each stress. The Z-score of each gene is presented using a color scale. The right side of each heatmap indicates gene ID with Arabidopsis gene symbol. (**b**) A Venn diagram of the number of shared DEGs between stresses. (**c**) Representative stress related GO terms in biological process. Bubble color indicates p-value (−log_10_ FDR); size indicates gene numbers of the DEGs in GO terms. Man, mannitol.

To quantify global gene expression patterns for multiple abiotic stresses, the mapped reads were calculated into read counts for the individual pepper genes. The distributions of all samples for normalized read counts were compared and are shown as a boxplot in Fig. 3a. These distributions were similar between the samples. A PCA analysis revealed that the first two PCs explained most of the variance, and samples from each treatment belonged to the same cluster with similar patterns (Fig. 3b). We further investigated the global gene expression profiles by performing analyses of the DEGs related to each abiotic stress and compared the results to those of the mock control^27^ (See the file “DEG result for each stress.zip” at figshare). As shown in Fig. 3c, the y-axis depicts the fold change in the log_2_-transformed data, and the x-axis represents the log_2_-transformed average counts per million reads (CPM). Upregulated DEGs are highlighted in red, whereas downregulated DEGs are shown in blue, with an adjusted p-value < 0.05 and FC > |2|. The gene expression patterns for the 30 top-ranked DEGs are shown in a heatmap (Fig. 4a). We identified a total of 12,494 DEGs shared and unique between stress treatments (Fig. 4b). To validate plant responses to each abiotic stress, GO enrichments were analyzed^27^ (See the file “GO enrichment analysis result.zip” at figshare) and we represented stress-responsive GO enrichments showing conserved and unique GO terms by comparison of each treatment (Fig. 4c). The distinctive patterns of gene expression and GO enrichments suggest that these data would be useful for comparing changes in gene expression for other abiotic stresses.

## Data Availability

Codes that were used for the RNA-seq data processing are available at figshare^27^. Software and their versions were described in Methods.
